# Revealing the micromechanisms behind semi-solid metal deformation with time-resolved X-ray tomography

**DOI:** 10.1038/ncomms5464

**Published:** 2014-07-18

**Authors:** K. M. Kareh, P. D. Lee, R. C. Atwood, T. Connolley, C. M. Gourlay

**Affiliations:** 1 Department of Materials, Imperial College London, Prince Consort Road, London SW7 2AZ, UK; 2 School of Materials, The University of Manchester, Oxford Road, Manchester M13 9PL, UK; 3 Diamond Light Source Ltd, Harwell Science & Innovation campus, Didcot OX11 0DE, UK

## Abstract

The behaviour of granular solid–liquid mixtures is key when deforming a wide range of materials from cornstarch slurries to soils, rock and magma flows. Here we demonstrate that treating semi-solid alloys as a granular fluid is critical to understanding flow behaviour and defect formation during casting. Using synchrotron X-ray tomography, we directly measure the discrete grain response during uniaxial compression. We show that the stress–strain response at 64–93% solid is due to the shear-induced dilation of discrete rearranging grains. This leads to the counter-intuitive result that, in unfed samples, compression can open internal pores and draw the free surface into the liquid, resulting in cracking. A soil mechanics approach shows that, irrespective of initial solid fraction, the solid packing density moves towards a constant value during deformation, consistent with the existence of a critical state in mushy alloys analogous to soils.

## Abstract (web summary)

Soil-like granular flow has previously been shown when deforming semi-solid metals. Here, the authors measure bulk and grain-level deformation in semi-solid alloys in three dimensions using X-ray tomography, exploring shear-induced dilation between 64–93% solid and finding hints of a critical state.

## Graphical abstract

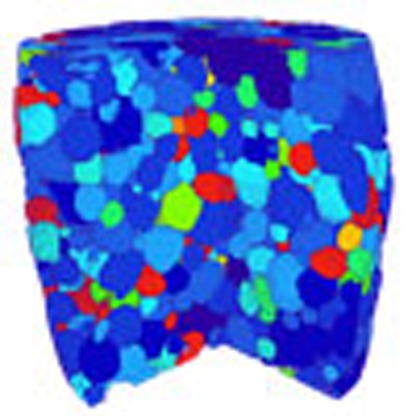

Solidification is the most direct route from liquid to engineering component, but difficulties remain in understanding and controlling casting defects. Many of the most damaging solidification defects develop once solidification has produced a solid network, at which point the permeability begins to decrease significantly and shrinkage and contraction strains become difficult to ‘feed’^1^,^2^. Understanding casting defects therefore requires a detailed understanding of deformation mechanisms at high solid fraction. Current interpretations of semi-solid alloys containing a solid network view the microstructure as either a continuous welded solid skeleton^3^, a network with partial cohesion^4^ or a cohesionless assembly of contacting grains^5^. The proposed micromechanisms vary from viscoplastic deformation of a porous solid skeleton^3^,^6^,^7^ (similar to a liquid-saturated sponge in compression) to the deagglomeration of a concentrated flocculated suspension^8^,^9^ (similar to dispersed clay slurries) or the granular rearrangement of quasi-rigid discrete grains with negligible cohesion^5^,^10^ (similar to a saturated particulate soil where force is transmitted across contacts between grains). Concerning the latter, it might be thought that partially solid alloys containing a solid network would not deform in this way; for example, metallic grains have a yield strength of only up to a few MPa in the solid–liquid two-phase region and grain–grain contacts can be cohesive due to the formation of solid–solid interfaces (grain boundaries), which would promote viscoplastic deformation of a porous solid without grain rearrangement. Furthermore, the concept of force transmission across a mechanical contact in solidification is complicated by the thermodynamics of interfaces in the mushy zone where either a solid–solid interface (a grain boundary) or a solid–liquid–solid interface (a liquid film) is stable^11^. Most of these interpretations of partially solid alloy rheology are based on bulk mechanical data coupled with *post mortem* microstructural analysis, and many of the proposed mechanisms have never been directly observed. Recently, time-resolved synchrotron radiography of thin samples has been applied to examine the flow behaviour of semi-solid alloys^10^,^12^,^13^. Some of these studies have revealed grains rearranging as independent bodies and exhibiting shear-induced dilation (Reynolds’ dilatancy)^10^,^13^ due to quasi-rigid grains pushing and levering each other apart during shear. However, these studies shear monolayers of grains that do not contain welds and may not reflect the deformation mechanisms of bulk three-dimensional (3D) samples.

Here, to directly identify the 3D grain-scale mechanisms of deformation and understand how they relate to the stress–strain response and lead to casting defects over a range of solid fractions, we have performed time-resolved tomography of uniaxial compression in Al–Cu alloys with globular (α-Al) grains at 64, 73, 86 and 93% solid. Bulk measurements in the form of stress–strain curves indicate the existence of a soil-like critical state, which is confirmed by the shear-induced dilation of the specimens at the scale of the grains. At high solid fractions, we show that this granular behaviour induces the formation of casting defects.

## Results

### Undeformed semi-solid microstructures

**Figure 1:**
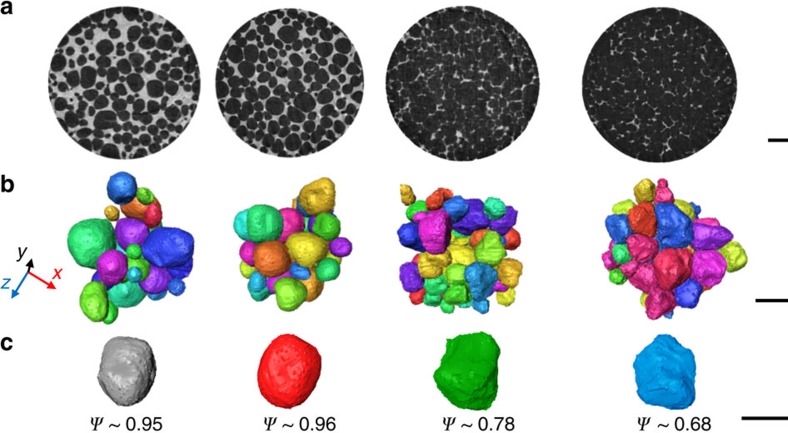
Undeformed semi-solid microstructures for nominal solid fractions of 64, 73, 87 and 93%. (**a**) Cross-sectional (*xy*) slices (scale bar, 1 mm), (**b**) 3D rendering of the separated grains (scale bar, 500 μm) and (**c**) typical grains from each specimen illustrating the decreasing sphericity, *ψ*, with increasing solid fraction (scale bar, 300 μm).

We begin by quantifying the initial semi-solid microstructures. In Fig. 1a, the solid appears dark and the liquid bright, with more crowded solid packing and more tortuous liquid regions at increasing solid fraction. Figure 1b shows 3D renderings of separated and filtered grains in 2 mm^3^ representative regions and Fig. 1c highlights the increase in surface topology with increasing solid fraction. In these samples, the mean grain sphericity decreases with increasing solid fraction, as the grains fill the interstitial space and acquire a more complex shape with increased surface area (quantified in Supplementary Fig. 1a and detailed in Supplementary Note 1). All specimens have average grain sizes within 15% of each other and the four grain populations are well approximated as self-similar (quantified in Supplementary Fig. 1), which is a consequence of the long-term isothermal semi-solid coarsening (for example, ref. 14) used in preparing the samples. All four specimens contain 10^−2^–10^−3^% porosity before loading (shown in Supplementary Fig. 2 and detailed in Supplementary Note 2).

### Bulk behaviour during *in situ* deformation

**Figure 2:**
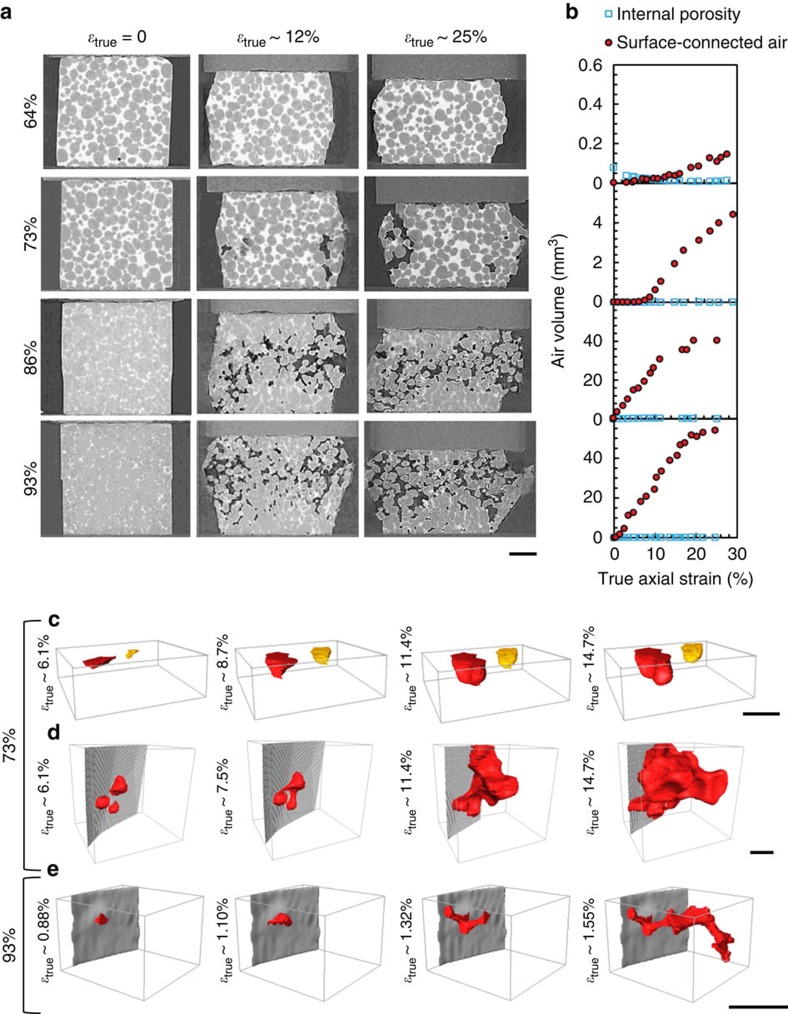
Uniaxial compression at 64, 73, 87 and 93% solid. (**a**) Transversal (*xz*) slices at increasing compressive axial strain (scale bar, 1 mm), (**b**) change in volume of internal voids and surface-connected voids with increasing strain and (**c**–**e**) 3D rendering of surface-connected meniscus development at 73 and 93% solid, respectively (scale bar, 300 μm).

Figure 2a shows vertical slices approximately halfway through the specimens at three stages during semi-solid uniaxial compression. A striking feature in Fig. 2a is that uniaxial compression causes porosity/cracking in the 73, 86 and 93% solid samples, which increases with increasing solid fraction and increasing ram displacement. We defined porosity as internal or surface connected and tracked it during deformation. Figure 2b shows that most porosity is surface connected (renderings of the porosity at different strains can be seen in Supplementary Fig. 3 and Supplementary Note 3). Figure 2c–e details the process by which air is drawn-in using 3D renderings of surface-connected pores at the sides of the 73 and 93% solid specimens. At 73% solid, the oxidised liquid surface is sucked into the sample both under the ram that is applying compressive load (Fig. 2c) and at the radial free surface (Fig. 2d). The two separate menisci in Fig. 2c develop directly underneath the ram and grow into the liquid during compression. The three radial menisci in Fig. 2d are initially pulled into the liquid before merging into a large surface-connected pore that propagates into the liquid between the grains, producing a complex pore with multiple radii of curvature. This mechanism is the same at 93% solid, but the packing of the solid is such that the propagation of a meniscus into the narrow liquid channel appears as cracking initiating from the surface (Fig. 2e). The drawing-in of menisci indicates that the liquid pressure is decreasing and the grains are moving apart. While this behaviour is common during tensile deformation and hot tearing^15^,^16^,^17^, it is counter intuitive during uniaxial compression and is not predicted by existing theories^4^,^8^. To clarify the underpinning mechanisms, we examined both stress–strain behaviours and discrete grain responses to load.

**Figure 3:**
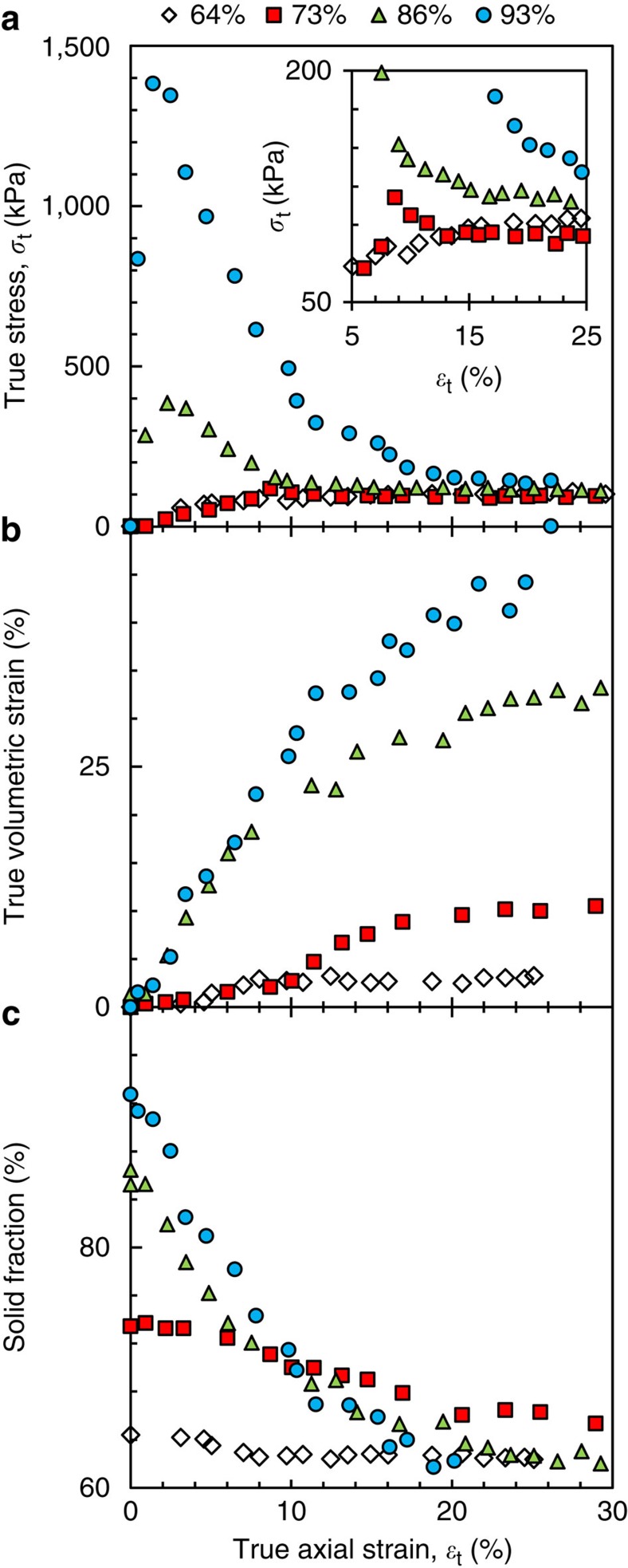
Bulk mechanical response at 64, 73, 87 and 93% solid. (**a**) True axial stress–true axial strain with the inset zooming in on the strain range at which all four stress responses reach a similar stress, (**b**) volumetric strain–true axial strain and (**c**) volume fraction of solid–true axial strain.

Figure 3a shows axial true stress–true strain curves where *in situ* imaging has been used to measure the true specimen area in contact with the moving ram. The 73, 86 and 93% solid samples all exhibit a peak whereas the 64% sample does not (see the inset in Fig. 3a), and the peak stress increases with increasing solid fraction. The samples that have a peak stress exhibit strain softening before a final period of deformation occurs at relatively low constant stress (115±17 kPa). Following granular mechanics, we define the strain with respect to the solid assembly rather than the whole material, such that a contractive volumetric strain occurs if grains move closer together and liquid/gas is expelled and a dilatational volumetric strain occurs if grains move apart and liquid/gas is drawn into the expanding interstitial spaces^18^. Here, the sum of the developing surface-connected porosity, internal porosity and the liquid phase was defined as the ‘interstitial fluid’ and the change in the volume of solid plus interstitial fluid was used to calculate the volumetric strain in Fig. 3b. When viewed in this way, all specimens undergo a volumetric expansion during deformation and the maximum volumetric strain increases with increasing solid fraction.

Since the volume of solid in each sample is near constant during isothermal deformation, and the volume of interstitial fluid increases during deformation, the solid fraction, defined as *g*_S_=1−(*g*_all porosity_+*g*_L_), decreases during compression. The solid fraction is plotted versus axial strain in Fig. 3c, showing that steady states seem to be reached for both the stress and the solid fraction. Importantly, the steady-state values of axial stress (115±17 kPa) and solid fraction (62.4±1.9%) are approximately the same for all samples. Note that this trend is only apparent when the *in situ*-measured true specimen contact area in each tomogram is used to calculate true stress. Thus, irrespective of the initial solid fraction, all samples have moved to a similar steady-state combination of solid fraction and axial stress, with the lowest solid fraction of 64% solid remaining within the 62.4±1.9%, which all samples reach during deformation. It is important to note, however, that this analysis does not account for variation between samples as it is based on one sample at each solid fraction, and that the significant porosity complicates the stress state and homogeneity.

Interesting insight can be drawn by comparing Fig. 3 with the typical response of a fully saturated particulate soil. In soil mechanics, the solid fraction (expressed via the void ratio, *e*=*V*_fluids_/*V*_solids_) moves towards a constant value during deformation. The soil then continues to deform at constant stress and constant void ratio, and is said to be at its ‘critical state’^18^. The similar behaviour between dense soils and the stress-strain response shown in Fig. 3 suggests that, with no confining pressure, the semi-solid alloys tested here have a critical state of 62.4±1.9% solid (*e*=0.602) and 115±17 kPa axial stress. Consistent with critical state soil mechanics theory, at solid fractions higher than 62.4±1.9% solid, the grain assembly undergoes macroscopic dilation to decrease its overall solid fraction and an alloy near the critical state deforms with negligible volumetric strain (as seen in Fig. 3). Testing whether semi-solid alloys follow other aspects of critical state soil mechanics theory is now necessary. For example, at solid fractions lower than 62.4±1.9% solid, and where a solid network still exists, the grain assembly is predicted to undergo macroscopic compaction to increase its overall solid fraction up to 62.4±1.9% solid, and the critical state is predicted to increase with the application of confining pressure.

### Discrete grain behaviour during *in situ* deformation

**Figure 4:**
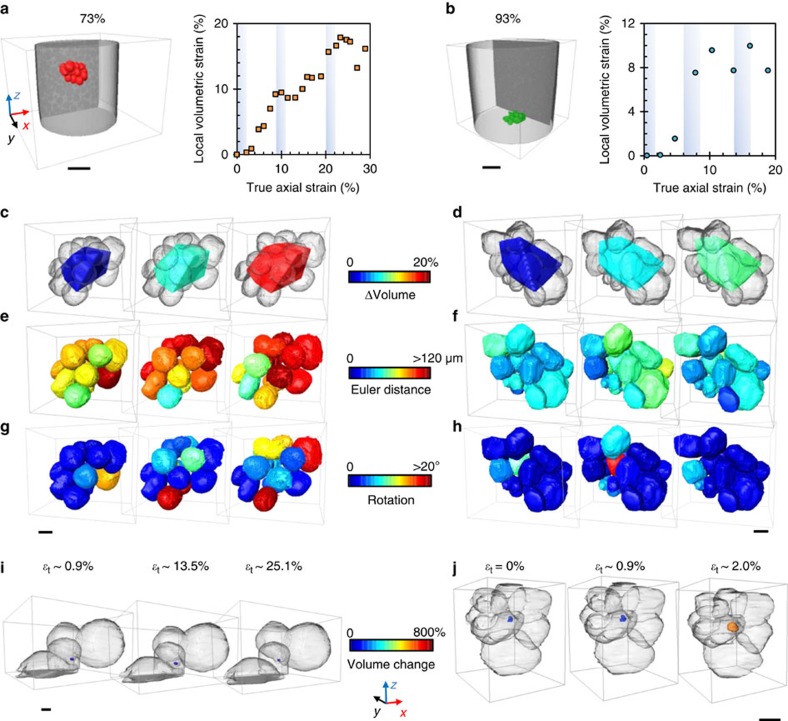
Dilation of 15- and 16-grain assemblies in continuous contact at 73 and 93% solid. Position and volumetric strain of each assembly for increasing axial strain at (**a**) 73% solid (scale bar, 1 mm) and (**b**) 93% solid (scale bar, 1 mm); 3D rendering of the polyhedron formed by the grain centroids at (**c**) 73% solid (scale bar, 300 μm) and (**d**) 93% solid (scale bar, 500 μm); Euler distance travelled by each grain per 2% incremental strain at (**e**) 73% solid (scale bar, 300 μm) and (**f**) 93% solid (scale bar, 500 μm); rotation of each grain per 2% incremental strain at (**g**) 73% solid (scale bar, 300 μm) and (**h**) 93% solid (scale bar, 500 μm); and internal porosity response at (**i**) 73% solid (scale bar, 100 μm) and (**j**) 93% solid (scale bar, 100 μm).

The micromechanisms leading to the macroscopic volumetric strains in Fig. 3 were investigated by studying the behaviour of individual grains during compression, in a manner similar to the study of the kinematics of sand grains imaged during uniaxial compression^19^,^20^. Within the resolution limit of this study, there is no statistically significant solidification or remelting of individual grains during an experiment (quantified in Supplementary Fig. 4 and detailed in Supplementary Note 4), and there is no detectable shape change of individual grains (quantified in Supplementary Fig. 5 and detailed in Supplementary Note 5). Grains can therefore be considered quasi-rigid and global deformation occurs by the rearrangement of grains, the flow of liquid and the motion of menisci. Sub-assemblies of 15 and 16 grains were then randomly selected for detailed analysis in 73 and 93% solid samples. 3D renderings of the sub-assemblies are shown in Fig. 4a,b. Note that these grains were in continuous contact during rearrangement and are from regions with no porosity at any strain, so that the in-flow or out-flow of liquid compensated for any changes in local solid packing. The volume of each local 15/16 grain assembly was defined by the polyhedron formed by the centroids of the grains and the development of polyhedron volume was used to calculate the volumetric strain plotted in Fig. 4a,b. The volumetric strain increases with increasing axial strains in both cases and the grain-level behaviour is shown in Fig. 4c,d, where the grains are rendered in grey and the polyhedron is coloured based on volume change. Fig. 4d–h shows the translation vector magnitude and grain rotation, respectively, and indicates that each grain is displacing independently, since neighbouring grains coloured identically at one increment often have different colour in the next and neighbouring grains travelling identical magnitudes often undergo a different rotation. From Fig. 4c–h it can be inferred that shear-induced dilation is due to quasi-rigid grains pushing each other apart as they translate/rotate independently under load, both at 73 and 93% solid. The dilatational volumetric strain and the resulting drawing-in of menisci are then emergent phenomena simply caused by the rearrangement of initially tightly packed quasi-rigid grains, and would not be expected if strain was only accommodated by viscoplastic deformation of the solid phase. The shear-induced dilation of grains/crystallites is also the origin of dilatant shear banding both in semi-solid alloys^5^ and in other systems such as soils^18^,^21^,^22^, rock^23^,^24^, magma^25^ and cornstarch slurries^26^,^27^,^28^.

### Defect formation at high solid fraction

**Figure 5:**
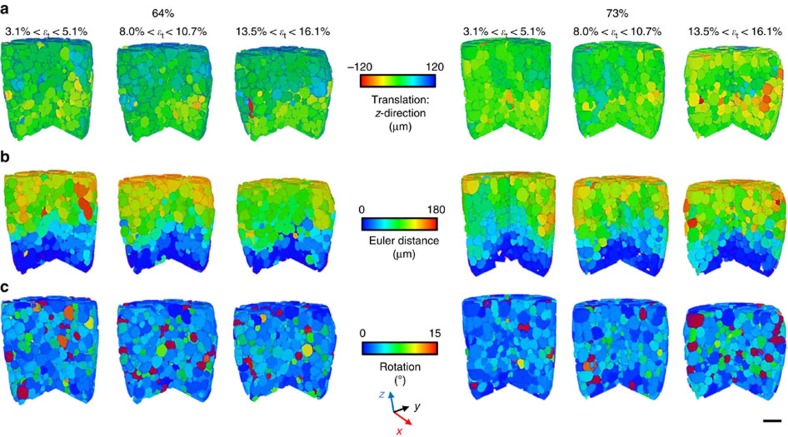
Discrete grain response of the whole specimen at 64% (left) and 73% (right) solid. (**a**) The translation of each grain in the *z*-direction after subtracting the *z*-component expected of homogeneous compression, (**b**) the Euler distances travelled by each grain and (**c**) the rotation of each grain. Each rendering is for a 2% incremental axial strain (scale bar, 1 mm).

At 93% solid, this shear-induced dilation causes not only surface cracking but also the significant opening of pre-existing internal porosity. Figure 5i,j shows 3D renderings of pre-existing gas-filled pores in their grain neighbourhoods. In contrast to the pore at 73% solid, whose volume change can be considered insignificant since it is at the limit of our resolution (detailed in Supplementary Fig. 6 and Supplementary Note 6), the pore at 93% solid opens under compressive strain with a volume increase of ~622% as grains are pushed apart. Other pores shown in Supplementary Fig. 7 also grow into the tortuous liquid channels between grains. Thus, during unfed compression at 93% solid, the expanding interstitial spaces during shear-induced dilation cause the local liquid pressure to drop and small internal pre-existing pores to grow due to the low permeability and lack of feed liquid. This highlights how shear-induced dilation can be another origin of defects in castings.

Figure 5 is an extension of the discrete methods in Fig. 4 to the specimen scale at 64 and 73% solid, and shows (i) grain translations in the normalized *z*-direction (that is, each measured z-translation minus that expected of homogenous uniaxial compression), (ii) the Euler distance (scalar translations) and (iii) the scalar rotation angle of each grain. The near-uniform displacement in Fig. 5a shows that the solid does not develop significant inhomogeneities during deformation at either solid fraction. In Fig. 5b,c, most grains translate or rotate by a different amount than their neighbours, indicating that the independent behaviour quantified in Fig. 4 extends to most grains in both specimens. Although the 64 and 73% solid samples exhibit a different stress–strain response (73% has a peak stress, 64% does not), the grain-level mechanisms are similar and the different macroscopic response is due to the amount of dilation required for grain rearrangement in the different initial solid fractions.

## Discussion

Semi-solid-processing models often assume that the peak stress is associated with the breaking of welds^7^,^29^,^30^. The sample preparation procedure used here (192 h coarsening at *T*_eutectic_+5 K) will have encouraged welded structures via coalescence ripening^31^, yet all grains moved as independent bodies throughout the deformation. This work shows that bulk stress rising to a peak is caused by the work done in pushing grains apart during shear-induced dilation and in drawing menisci into the specimens. Other models predict that semi-solid compression is equivalent to the compression of a liquid-saturated sponge^6^,^32^. Here, the opposite of densification has been measured, with interstitial fluid drawn into the entire specimen via closely-packed grains pushing themselves apart as they rearrange under compressive load. Future work needs to assess whether this behaviour is significantly altered by higher strain rates, different crystal morphologies and the availability of feed liquid.

This *in situ* 3D grain-scale study has revealed that globular semi-solid metal microstructures from 64 to 93% solid all deform as near-cohesionless granular materials, despite containing tightly packed assemblies of soft partially cohesive grains. The shear-induced dilation of these structures leads to what seems to be a critical state of 62.4±1.9% solid at 1 atm (as defined by critical state soil mechanics^18^), and can create casting defects by driving pore opening and/or crack propagation.

## Methods

### Casting and globularisation

Al–(8–15)Cu (wt.%) alloys were melted, grain refined with 0.01% Al–5Ti–B, degassed and gravity die cast to produce an equiaxed dendritic microstructure of average grain diameter 160 μm. Ø10 mm cylindrical rods were machined from the casting and given a semi-solid heat treatment for 192 h at 5 °C above the eutectic temperature to generate the large-scaled globular microstructures in Fig. 1. Masses and alloy compositions are given in Supplementary Tables 1 and 2. Details of the casting and the globularisation processes are given in the Supplementary Methods (subsections A and B).

### *In situ* experiments

Heating and loading were applied and measured by a bespoke furnace and tension-compression rig on the JEEP (I12) beamline at the Diamond Light Source (UK). Specimens were placed in a boron nitride cylindrical cup with inner diameter=8 mm, wall thickness=1 mm and height=10 mm and isothermally held at various solid fractions. Uniaxial compression was applied at constant ram speed of 5 and 1 μm s^−1^ per constant strain rates of 1 × 10^−3^ s^−1^ and 2 × 10^−4^ s^−1^ using a BN-coated pyrophyllite ram during continuous *in situ* image capture. A 53-keV incident X-ray beam was used while rotating the specimen at 15° s^−1^, and projected images were recorded every 1° with an exposure time of 33 ms, using a 500-μm-thick Lu:Ag:Ce scintillator and Phantom v7.3 camera. The working field of view was 9.8 × 7.3 mm with 12.22 μm resolution. Details of the *in situ* experiments are given in the Supplementary Methods (subsection C).

### Image processing

A reconstruction algorithm based on the filtered back projection method^33^ was used to reconstruct the 3D volumes. All volumes were aligned within the same coordinate system using a normalized mutual information metric similarity measure. Grains were segmented using a region-growing thresholding method followed by a watershed algorithm (both commercially implemented), their centroids tracked using a diffusion method^34^ and changes in their morphology (volume, surface area, shape) quantified using specific shape factors. Discrete grain translations were measured by centroid tracking, while rotation used a grain-based image correlation approach based on the highest normalized correlation coefficient. Quantification of internal and external porosity relied on Otsu-based thresholding. All volumes were processed using imaging packages Avizo (Visualization Science Group, France), ImageJ (US NIH, USA) and MATLAB 7.1 (The Mathworks, USA). A step-by-step illustration of the image processing is provided in the Supplementary Methods (subsection D, Supplementary Figs 8–15).

## Author contributions

C.M.G. and P.D.L. supervised the project, T.C. and R.C.A. modified the beamline for the experiments and K.M.K., R.C.A., P.D.L. and C.M.G. designed and acquired the experimental data. K.M.K. carried out the analysis and wrote the manuscript with C.M.G. and contributions from P.D.L.

## Additional information

**How to cite this article:** Kareh, K. M. *et al.* Revealing the micromechanisms behind semi-solid metal deformation with time-resolved X-ray tomography. *Nat. Commun.* 5:4464 doi: 10.1038/ncomms5464 (2014).

## Supplementary Material

Supplementary Information Supplementary Figures 1-15, Supplementary Tables 1-3, Supplementary Notes 1-6, Supplementary Methods and Supplementary References
