# Supplementary material for: Revealing the micromechanisms behind semi-solid metal deformation with time-resolved X-ray tomography
Source: Nat Commun. 2014 Jul 18;5:4464. doi: 10.1038/ncomms5464 (PMC4109016; doi:10.1038/ncomms5464)
Supplement: Supplementary Information — Supplementary Figures 1-15, Supplementary Tables 1-3, Supplementary Notes 1-6, Supplementary Methods and Supplementary References [file ncomms5464-s1.pdf]

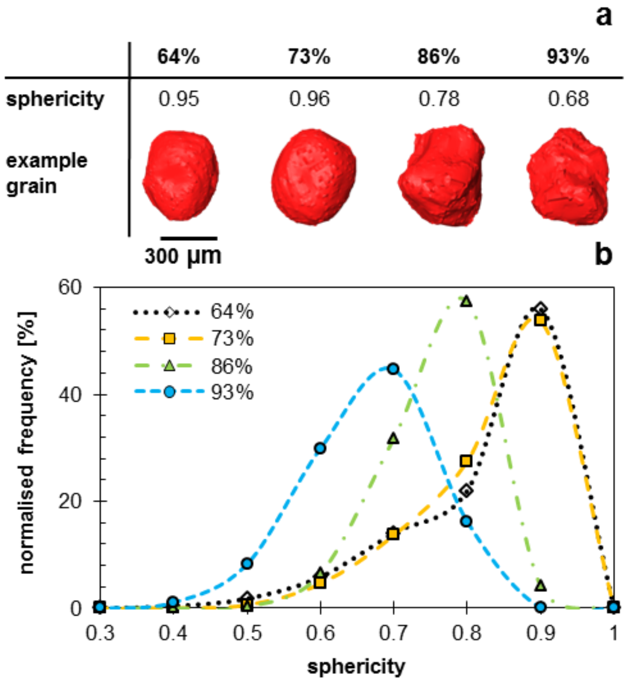

Supplementary Figure 1. Grain shape and sphericity distribution at the four solid fractions investigated. a) Typical grains from each specimen illustrating the change in shape associated with decreasing sphericity and shown in Fig.1, b) sphericity distribution for the four solid fractions.

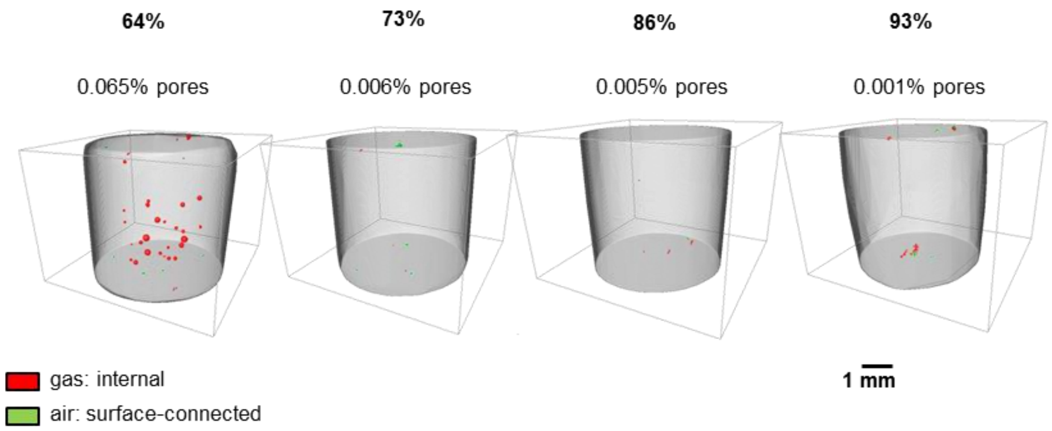

Supplementary Figure 2. Pre-existing porosity in the four undeformed semi-solid specimens. Porosity is red when it is inside the samples and green when it is touching the sample surface.

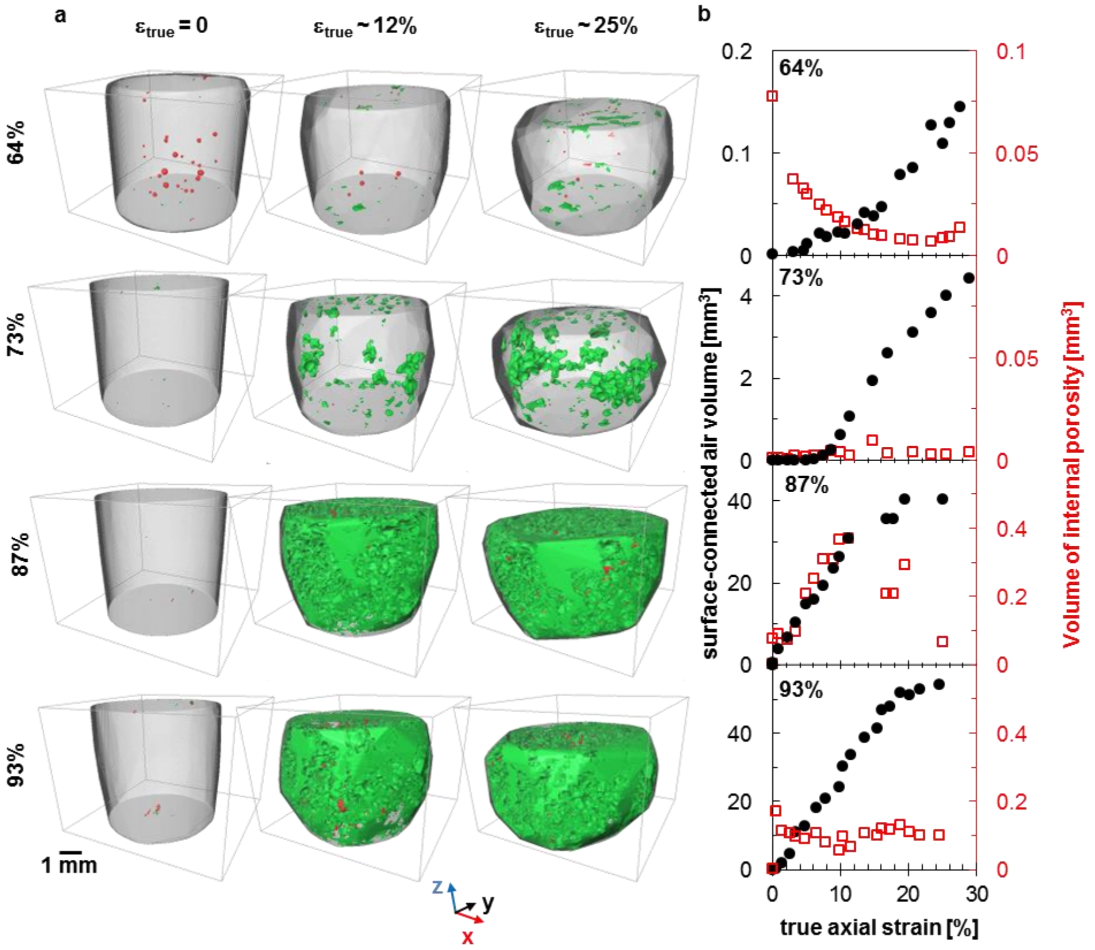

Supplementary Figure 3. Internal and surface-connected porosity evolution during deformation. Change in volume of internal voids and surface-connected voids with increasing strain during uniaxial compression plotted in c) with b) 3D renderings of the internal voids (red) and surface-connected voids (green) for all four solid fractions. Note the different pore volume axis for each sample.

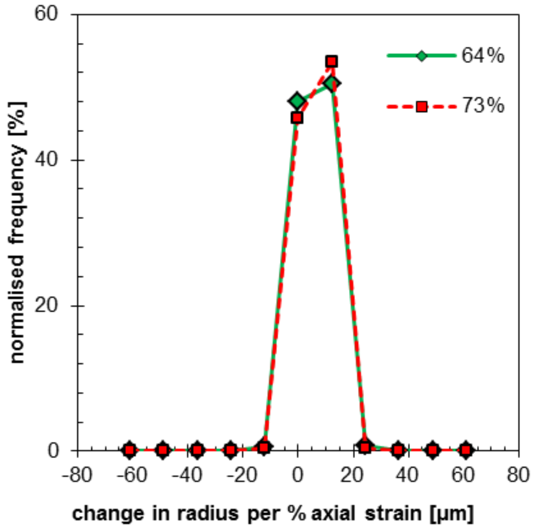

Supplementary Figure 4. Grain volume change during the experiments. Change in radius per 2% strain increment at solid fractions of 64% and 73%.

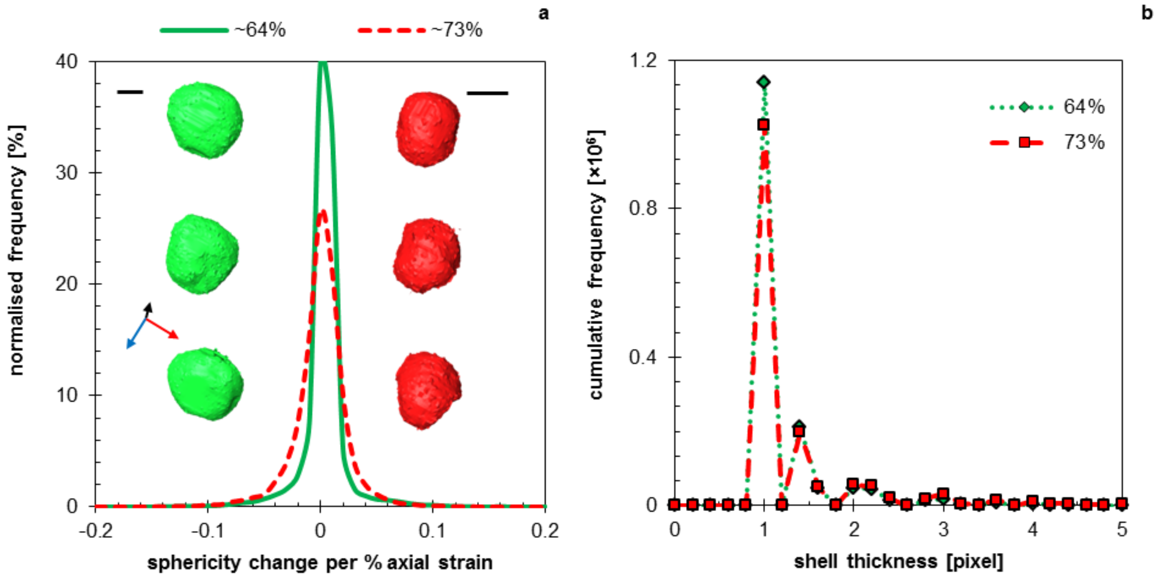

Supplementary Figure 5. Grain shape change during the experiments. a) sphericity change per 2% strain increment at solid fractions of 64% and 73% (scale bars are 120 um) — the sphericity change of all the grains at both solid fractions during each experiment is within ±5%; b) for a random sampling of 50 grains, a histogram of the cumulative thickness of the change in shape of each grain for solid fractions of 64% and 73% — shell change is between 1 and 2 pixels for almost all grains.

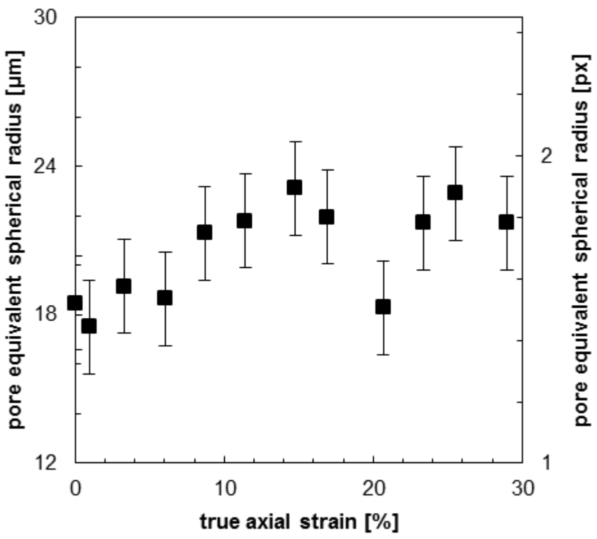

Supplementary Figure 6. Evolution of the volume of a sample pre-existing pore at 73% solid. Change in equivalent spherical diameter and in the volume of the pore illustrated in Fig.4 at 73% solid, showing overall change in the pore is very small: while there is a slight increase in the size of the pore this corresponds to an increase of less than 3 pixels in equivalent sphere radius, which is near the limit of our resolution.

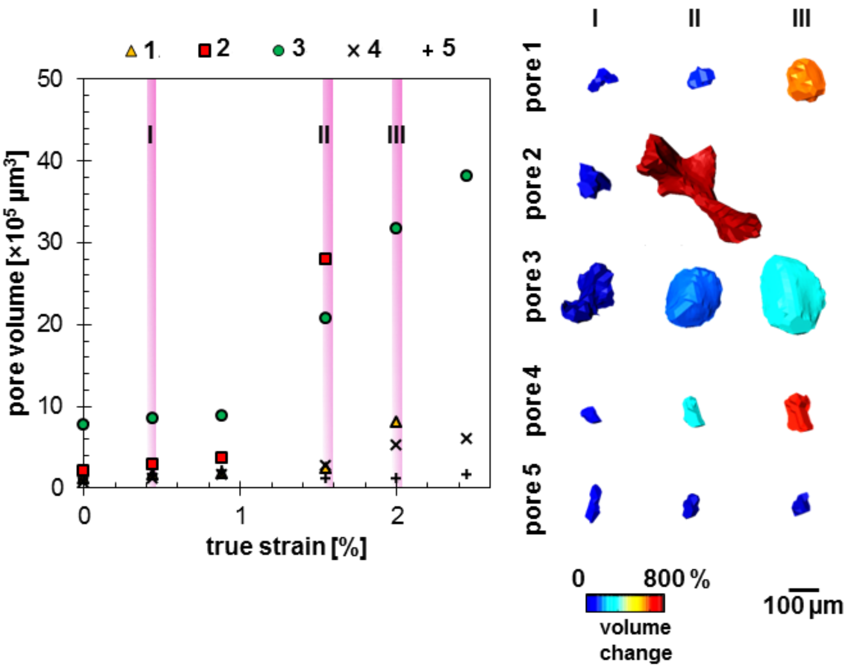

Supplementary Figure 7. Evolution of the volume of pre-existing pores at 93% solid. a) plot of the change of pre-existing pore volumes with increasing strain; b) 3D rendering of the pores tracked in a).

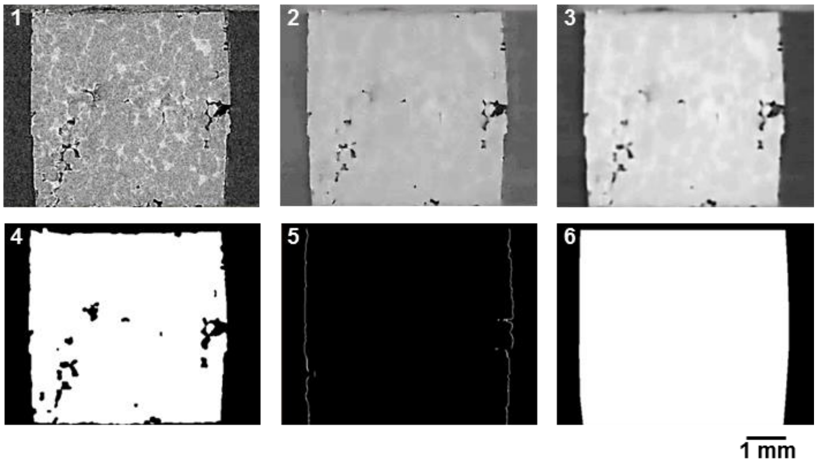

Supplementary Figure 8. Specimen masking process. 1) shows the original volume, 2) the non-linear diffusion filtered volume to smooth the internal microstructure of the specimen, 3) the convolved filtered volume using a 10×10×10 ellipsoid, 4) the thresholded convolved volume, 5) the 2D edge volume with the ram removed, and 6) the final mask obtained via a convex hull algorithm run on the edge volume. The specimen here is at g_S ~87% and ε_T~0.9%.

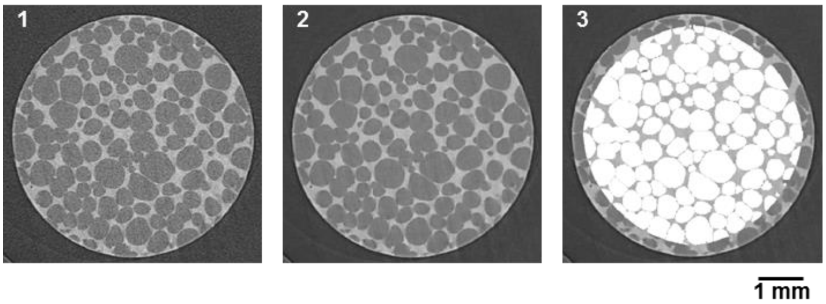

Supplementary Figure 9. Smoothing and edge-preserving filter as a prelude to further processing. 1) shows a reconstructed slice in the *xy* direction, 2) shows the same slice after a non-linear diffusion filter has been applied to the entire volume, 3) showcases further processing (solid thresholding and watershed—detailed below) as a result of the non-linear diffusion filtering. The specimen here is at g_S ~74% before deformation.

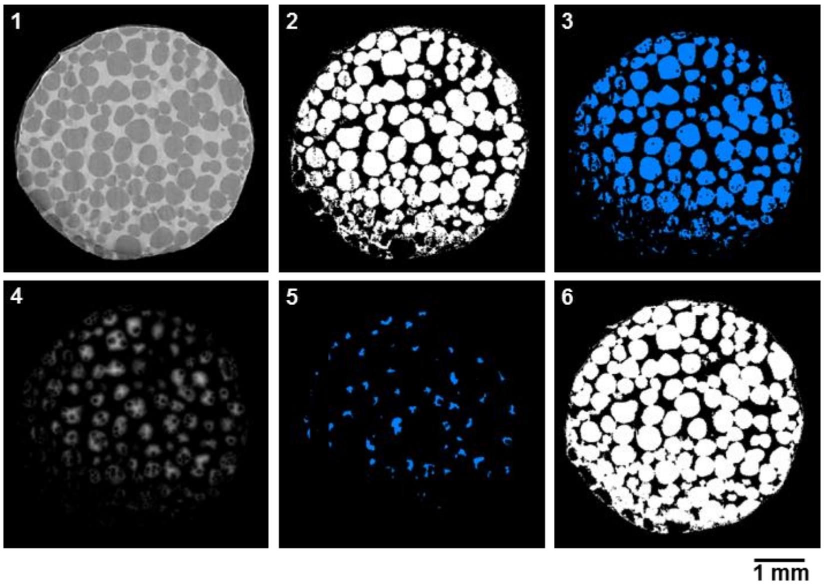

Supplementary Figure 10. Thresholding of the solid phase using a region growing algorithm, which requires a seed volume. 1) shows the original volume, 2) the global Otsu thresholding of the solid, 3) a 1-pixel erosion of the solid, 4) a distance map of the eroded thresholded solid, and 5) maxima of the distance map. These maxima act as the seeds for the region-growing algorithm whose result can be seen in 6). The specimen here is at g_S ~64% and ε_T~13.5%.

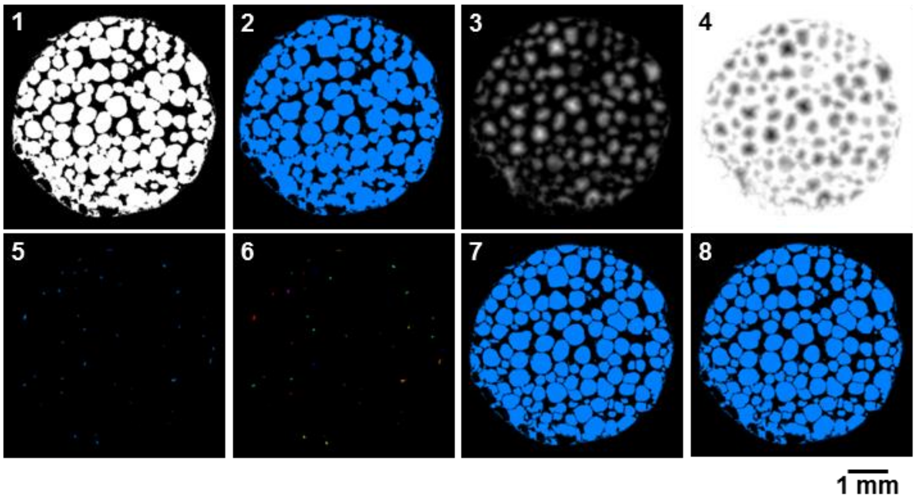

Supplementary Figure 11. Globule separation using the watershed technique. 1) shows the binarised solid phase, 2) the solid phase with the holes filled (difference noticeable in the bottom left), 3) the distance map of the binarised solid, 4) the inverse of the distance map to be used as the first watershed input, 5) the distance map maxima, 4) the labelled distance map maxima to be used as the second watershed input, 7) the separated globules and finally 8) the volume-filtered final separated globules. The specimen here is at g_S ~64% and ε_T~13.5%.

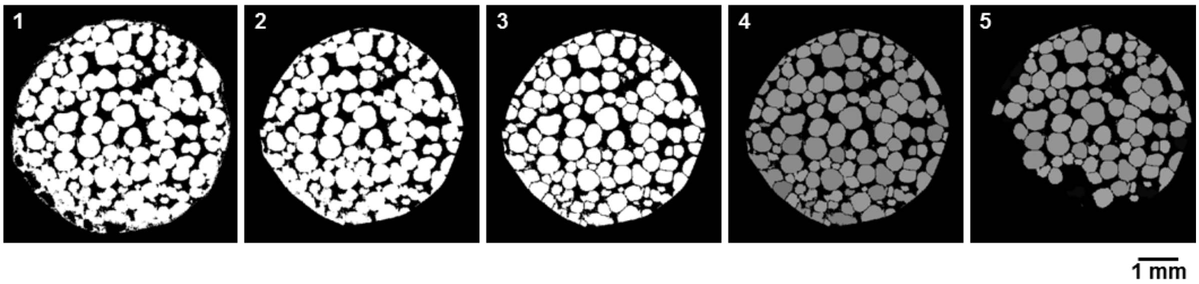

Supplementary Figure 12. Illustration of the globule tracking process. 1) shows the region-grown thresholded solid, 2) shows the eroded solid, 3) shows the separated and filtered globules, 4) shows the labelled globules, and 5) shows the tracked and labelled globules. The specimen here is at g_S ~64% and ε_T~13.5%.

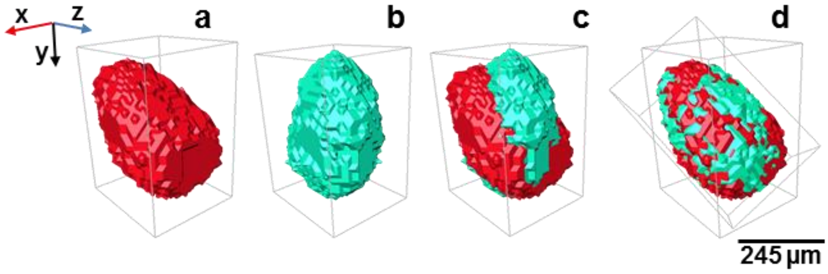

Supplementary Figure 13. Rotation using image correlation. (a) grain at ε_T~8.7%, (b) grain at ε_T~11.4%, (c) overlapping grains before registration, (d) overlapping grains after registration.

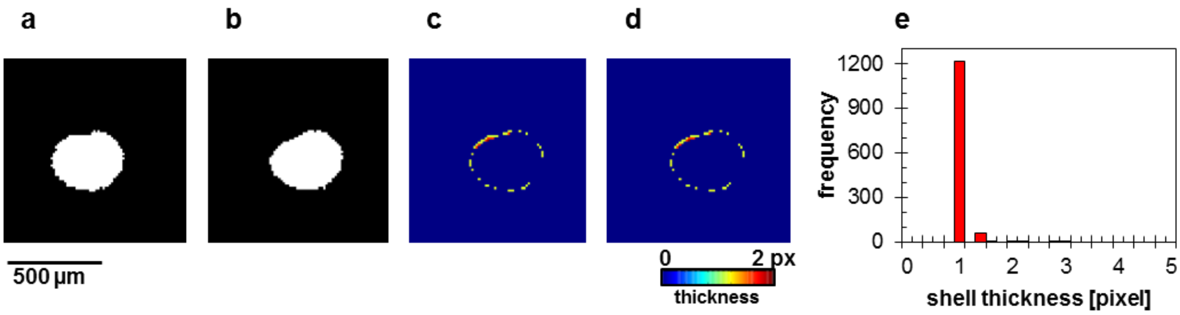

Supplementary Figure 14. Shell thickness calculation. (*xy*) slices of a) the grain at the previous timestep, b) the grain at the actual timestep, c) the distance map of the non-overlapping voxels, d) the voxels in common with the distance map and the dilated voxels of the grain at the actual timestep, and e) the frequency of the thickness values.

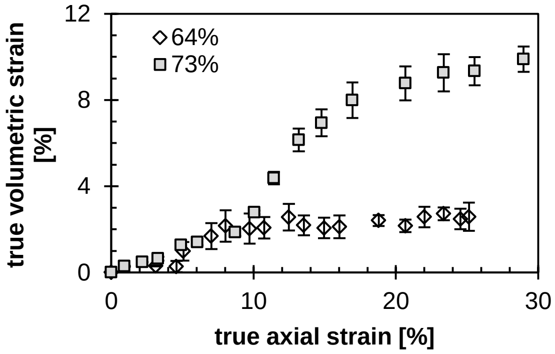

Supplementary Figure 15. Convex hull calculation comparison. True volumetric strain during compression experiments at $fs \approx 64\%$ and 73%. The volumetric strains plotted are the average of the centroid and specimen edge volumetric strains. The error bars represent the spread covered by both values.

Supplementary tables

	CP-Al [g]	Al-50wt.%Cu [g]	TiB_2 [g]
**Al-15wt.%Cu**	1931	828	27.8
**Al-8wt.%Cu**	1302	248	15.56

Supplementary Table 1. Weights of materials for wedge casting.

wt.% Cu	Zr	Zn	Cu	Fe	Cr	Ti	Si	Mg	Mn
**15**	0.023	0.014	14.650	0.13	n.d.	0.042	0.010	0.069	0.013
**8**	0.023	0.011	8.340	0.14	n.d.	0.043	0.019	0.068	0.009

Supplementary Table 2. Alloy compositions. Quantitative XRF results of % w/w alloying elements (n.d = not detected, referring to < 0.0002 wt.%).

**Nominal wt. Cu**	**Recorded holding temperature**	**Calibrated holding temperature**	$g_{s_{image}}$	$g_{s_{Lever}}$	$f_{s_{Lever}}$
[%]	*[°C]*	*[°C]*	*[%]*	*[%]*	*[%]*
15	531.1 ± 2.3	580.6 ± 2.3	64.38	62.21	59.99
15	512.8 ± 1.7	562.4 ± 1.7	73.43	73.60	71.08
8	539.2 ± 1.4	588.7 ± 1.4	86.43	87.01	86.12
8	518.6 ± 1.7	568.1 ± 1.7	92.70	94.59	93.96

Supplementary Table 3. Solid fractions deformed *in situ*.

Supplementary notes

Supplementary Note 1: As shown in Fig.1 of the article and reproduced here, Supplementary Figure 1(a) shows a typical grain from each undeformed specimen. At higher solid fraction, grains have increased surface topography characterised by protrusions and dips, which are most evident in the 93% solid specimen. The sphericity distributions are shown in Supplementary Figure 1(b) for all four specimens. The 64% and 73% solid samples have similar sphericity distributions and the sphericity decreases with increasing solid fraction. The decrease in sphericity is related to grains filling the space between grains at high solid fraction. Here, higher packing is thus synonymous with a change to an increasingly complex crystal morphology accompanied by interstitial liquid channels that are smaller and more tortuous.

Supplementary Note 2: All four semi-solid specimens contain porosity before load is applied as shown and quantified in Supplementary Figure 2. Specimens at 73%, 86% and 93% solid have volumes fraction of porosity of the same magnitude of 10^{-3}% while the specimen at 64% solid has a volume fraction of initial porosity of 10^{-2} %.

Supplementary Note 3: The evolution of porosity during deformation at all four solid fractions is rendered in Supplementary Figure 3(a) and quantified in Supplementary Figure 3(b), which is a reprint of Fig.2(b) with detailed scaling of the secondary axes.

Supplementary Note 4: *Grain shape.* To investigate whether grains are deformed during uniaxial compression, the change in sphericity of each grain was studied between tomograms. Supplementary Figure 4(a) shows histograms of the change in sphericity per 2% percent axial strain increment for the 64% and 73% solid samples. The sphericity change of all the grains at both solid fractions during each experiment is within $\pm$5%. In addition, Supplementary Figure 4(b) shows that the accumulated surface change of 50 randomly selected grains in each specimen over the entire experiment duration is, only between 1 and 2 pixels for almost all grains. The apparent shape change of the grains is thus within the detection resolution of our experiments. Therefore, within the resolution limit of this study,

the grains can be considered quasi-rigid and deformation occurs by the rearrangement of grains, the flow of liquid and the motion of menisci.

Supplementary Note 5: *Grain volume.* To explore any solidification and/or melting of the solid α-Al grains during deformation, the change in radius of individual grains was computed per 2% axial strain increments and is shown in Supplementary Figure 5. The radii change ranges from less than -10 μm to 20 μm, which is equivalent to a range of -1 to 2 pixels. Therefore, within the resolution limit of this study, there is not any significant solidification or remelting of the individual grains, and deformation is well-approximated as isothermal.

Supplementary Note 6: Changes in the internal pores were quantified in the specimens at 73% and 93% solid in order to compare and contrast pore behaviour at low and high solid fractions. In order to temporally resolve pore evolution at this solid fraction, the usage of the volume deformed at 1 μm s^{-1} is necessary. Only true strains of 0-2.5% were examined, i.e. the first moments after the start of deformation. The detailed behaviour of a number of randomly selected pre-existing pores was explored during uniaxial compression at 93% solid. Nine pre-existing pores were identified and tracked, and the evolution of five of them shown in Supplementary Figure 7. The selected pore in Fig.4 of the letter is 'pore 1' here. Pre-existing pores overwhelmingly open up under strain. The evolution of pores 1 to 5 is plotted in Supplementary Figure 7(a) and rendered in Supplementary Figure 7(b) for increasing strain. Pore shape develops from non-spherical to either a rounder morphology (pore 1, pore 3, pore 4) or they elongate during growth into the tortuous liquid channels between grains (pore 2). Only one pore seems to remain at a near-constant volume (pore 5 in Supplementary Figure 7(a)), but its 3D rendering (Supplementary Figure 7(b), last row) shows it actually grows under strain and develops from a more tortuous to a more elliptical shape.

In contrast, the specimen at 73% solid has very few internal pores, which are small and near the surfaces (see Supplementary Figure 2). The behaviour of the selected pore in Fig.4 of the letter is shown in Supplementary Figure 6. It can be seen that there is a slight increase in the size of the pore but this corresponds to an increase of less than 3 pixels pixels in equivalent sphere radius, which is

near the limit of our resolution. The behaviour of this selected pore is qualitatively representative of all pores in the 73% solid sample where any increase in pore size is considerably smaller than that during uniaxial compression at 93% solid.

Supplementary methods

A. Casting. Three alloy wedge castings with two compositions, Al-8Cu (wt.%) and Al-15Cu (wt,%), were produced for this work. Binary hypoeutectic Al-xCu (wt.%) alloys were chosen because of the suitable solid-liquid contrast they provide under X-rays. This is due to a combination of absorption contrast and density contrast, with a copper-rich liquid phase denser than the solid α-Al crystals because of the rejection of copper into the liquid during solidification. The castings were produced with commercial purity Al (Hydro Norsk ASA, Norway) that was heated using a resistance furnace in a clay-graphite crucible monitored by a K-type thermocouple. Once liquid, an Al-50 wt.% Cu master alloy (LSM, Rotherham, UK) was added to produce an Al-15 wt.% Cu or an Al-8wt.%Cu composition. When the melt reached 770°C, it was stirred thoroughly and degassed using Ar for approximately 15 minutes. TiB_2 grain refiner was then added in the form of Al- 5Ti-B rods (LSM, Rotherham, UK) and stirred thoroughly. The melt was then cast into the steel rectangular wedge mould detailed in reference [1] coated in Foseco Dycote (UK) and preheated to 150°C. The casting had dimensions 200×150×20 mm at the top and 200×150×12 mm at the bottom. Metal weights are given in Supplementary Table 1. Cast alloy composition as determined by X-ray fluorescence is shown in Supplementary Table 2.

B. Globularisation. Cylindrical rods of 13 mm and 10 mm in diameter were machined from the castings and heat-treated in the semi-solid state using a WHT6/30 forced-air convection oven (Lenton, London, UK) to create globular α-Al grains for the *in situ* studies. The holding temperature was set to 553 ± 1°C (corresponding to approximately 4.8 ± 1°C above the eutectic temperature), and was monitored using a K-type calibrated thermocouple. Specimens were placed in Al_2O_3 tubes in a borosilicate glass container and were heat-treated for 8 days. At the end of the heat treatments, samples were cooled in air. The heat-treated cylinders were subsequently re-heated to approximately 550 ± 1°C in a tube furnace under low vacuum (approximately 1.3 Pa) for 5 hours to reduce porosity. The cylinders were then machined to ∅5×5 mm cylinders for compression experiments.

C. *In situ* experiments. All fast tomography was conducted on the Joint Environmental, Engineering and Processing beamline (JEEP, I12) at the Diamond Light Source synchrotron in Harwell, Oxfordshire. For *in situ* semi-solid deformation, a bespoke tension-compression rig (University of Manchester, UK) with a 500N ± 0.1N load cell, a continuous ram rotation over ± 0.001°, and a displacement range of 150 mm ± 200 nm was used[2]. Two-part boron nitride cells were designed for compression and extrusion. The temperature was controlled by a 1000°C PID-controlled furnace (University of Manchester, UK[2,3]) mounted on the tension-compression rig.

Semi-solid microstructures were created by heating specimens above the eutectic temperature and holding them isothermally for five minutes before the deformation proceeded. During the semi-solid heat treatments of 8 days it can be assumed that concentration gradients will have equilibrated, and the solid fraction can thus be calculated from using the Lever approximation. After deformation, the furnace was switched off and specimens allowed to cool in air.

The heat treatment and imaging conditions were optimised to obtain a microstructure and imaging field-of-view best suited both to (i) the JEEP beamline at the Diamond Light Source and to (ii) a time-resolved granular study of semi-solid alloy deformation. All experiments were done with a monochromatic beam of 53 keV because it was the lowest available energy at the beamline at the time of each experiment.

The solid sample was placed in a boron nitride cylindrical cup with ID = 8 mm, wall thickness = 1 mm, and height = 10 mm. It was compressed using a boron nitride coated ∅ = 7 mm cylindrical ram. Compression was investigated in Al-15Cu and Al-8Cu (wt.%) at various temperatures above the eutectic temperature at constant ram speeds of 5 µm s^{-1} and 1 µm s^{-1}.

For image capture, the specimen was continuously rotated at 15° s^{-1} and projected images were recorded every 1° with an exposure time of approximately 33 ms on an 800 × 600 CMOS chip on a Phantom v7.3 (VisionResearch) camera after being converted from X-rays to visible light using a 500 µm thick LuAg:Ce scintillator. The working field of view was 9.2 × 6.1 mm with a voxel size of

12.22 µm. This resulted in 360 projected images per tomogram, with 60 tomograms of 12 s each captured back-to-back during the experiment.

Temperatures were measured using a thermocouple in contact with the bottom of the boron nitride container. All experimental temperatures cycle within a 5°C window and affect both the theoretical mass and volume solid fraction (f_s and g_s respectively) by less than 1.5%. In this case, the theoretical mass fraction was estimated using the lever rule and a linear approximation of the liquidus curve, while the volume fraction used the conversion from f_s to g_s using liquid and solid densities as determined by Poirier and Ganesan[4]. The thermocouple readings were calibrated to give the temperature of the semi-solid sample by comparing the measured temperature in the container below the sample with the solid fraction measured by tomography. The calibrated temperature data for all samples is given in Supplementary Table 3.

An in-house reconstruction algorithm based on the filtered back projection method[5,6] was used to reconstruct the 3D volumes from the 2D transmitted images.

D. Imaging. Details of the steps undertaken during each phase of the image processing of this work are presented below, generally in their chronological order. All filters are three-dimensional unless otherwise mentioned.

Normalisation. All reconstructed volumes in one experiment were normalised to a reference volume, which consisted of a volume acquired before deformation at the beginning of the experiment. All normalisation in this work was linear.

Registration. In order for features to be tracked, the volumes must have a common coordinate system. They were thus each registered using an affine transformation matrix obtained via the normalised mutual information metric similarity measure[7]. This involves 3D rigid body transforms, whereby a volume is rotated, translated and scaled to a reference volume in order to align the objects within them based on voxel intensity similarity. Due to the large deformation applied to each specimen, each volume was registered to the volume before it using a region of interest which did not

undergo significant physical change as a reference. All transformed volumes were interpolated using a Lanczos low-pass filter approximation resampling method.

Masking. Masking is needed order to use intensity-based thresholding in terms of the phases present in the specimen. As can be seen in Supplementary Figure 8(1), the ram has similar greyscale intensity to the solid phase while the entrained air in the specimen has the same intensity as the air outside the specimen. By smoothing the internal microstructure of the specimen and making the specimen edges more prominent using a non-linear diffusion filter followed by a convolution using a 10×10×10 pixel ellipsoid (Supplementary Figure 8(2) and Supplementary Figure 8(3)), a preliminary mask can be obtained using global thresholding (Supplementary Figure 8(4)). As a result of the specific compression experiment undertaken in this work, a large percentage of the porosity present in the specimens is entrained air, which is therefore connected to the surface of the specimen. While there are multiple algorithms dedicated to closing this sort of feature, the large size of these specific voids precluded that option. Instead, the remnants of the ram were manually removed before the edges of the convolved specimen were isolated using a 2D Sobel edge detection algorithm (Supplementary Figure 8(5)). Their edges were used as inputs in order to compute a convex hull around the specimen (Supplementary Figure 8(6)). This allowed for the small air menisci at the surface of the specimen to be accounted for before they develop into large air voids.

Smoothing. Whatever data is contained within the mask can be analysed after masking. In order to proceed with binary processing, the original datasets must be smoothed to remove acquisition noise, which can be seen in Supplementary Figure 9(1). This work used an edge-preserving smoothing filter modelled on a non-linear diffusion process. After pre-filtering the data with a Gaussian filter, the grey level differences between neighbouring pixels were smoothed depending on the image gradient, resulting in Supplementary Figure 9(2): the inherent graininess of the reconstructed image was removed, while the edges of the specimen and the solid globules remained sharp. Supplementary Figure 9(3) shows further processing on this specific dataset (detailed below) and the good correlation between the binarised result and both the original data and the smoothed data.

Thresholding. All data was binarised using two methods: global thresholding using Otsu's method, and region-growing thresholding. Otsu thresholding involves the histogram shape, where pixels are assigned to different classes via the minimisation of the variance within each class. Region-growing thresholding, on the other hand, determines whether pixels neighbouring an initial seed point belong to the region being grown via the usage of a specific empirically-determined threshold criterion. Region-growing thresholding was used to isolate the solid phase of each volume (Supplementary Figure 10(6)) with seeds determined using the distance map of the solid phase after Otsu thresholding (Supplementary Figure 10(4), Supplementary Figure 10(2) and Supplementary Figure 10(5) respectively).

Air and other gases generally enjoys good absorption with the other heavier phases. In addition, the voids in all the specimens presented darker edges caused by phase contrast. Voids were thus thresholded using Otsu's method. The region-grown solid phase and the voids were then combined and subtracted from the mask to provide the liquid phase.

Watershed grain separation. Separation of the solid phase into individual globules was necessary to study granular behaviour. This was done using a watershed algorithm with steps detailed in Supplementary Figure 11. The region-grown solid was first filtered to fill holes and produce a distance map (Supplementary Figure 11(2) and Supplementary Figure 11(3)). The maxima in that distance map indicate the basin markers, i.e. the most inner regions within globules, which need to be labelled (Supplementary Figure 11(5) and Supplementary Figure 11(6) respectively). The maxima are not filtered or altered at this stage. The inverse of the distance map and the basin markers (Supplementary Figure 11(4) and Supplementary Figure 11(6) respectively) are then used as input for the watershed by flooding, which separated the globules (Supplementary Figure 11(7)). The separated globules were finally filtered by volume to remove voxel-sized artifacts (Supplementary Figure 11(8)). The cut-off volume used was 500 pixels^3, which is equivalent to globules with a diameter of approximately 120 μm.

Erosion. The nature of synchrotron experiments caused some artifacts at the edge of the specimens, mostly in terms of a greyscale gradient that darkened some regions near the edge. This is visible in Supplementary Figure 10(1), where global thresholding did not yield good results in the bottom left of the specimen. This gradient varied from tomogram to tomogram and grew worse with increased strain. Removing this sort of evolving artifact is manual and the process would have had to be modified for each volume. As a result, after grain separation, the mask of each volume was eroded in 2D in the *xy* direction by 20 pixels (244 µm), which is approximately half an average grain diameter in order to remove the problematic regions from subsequent processing. This is illustrated in Supplementary Figure 12(3). The grains are then individually labelled (Supplementary Figure 12(4)) and tracked using the position of their centroids.

Feature tracking.

Globules. Only globules that could be tracked for the entire duration of the experiment were analysed. Globules were allowed to 'blink' once, i.e. disappear in one tomogram during the tracking to grant some margin of error in terms of the watershed separation. The maximum distance L the centroids were allowed to move was 22 pixels, which is equivalent to 269.5 µm or approximately two-thirds of a globule diameter. Linking centroid locations to trajectories was done using labelled centroids proximity at different time steps [8]. A main limitation of linking centroid distribution to trajectories is that centroids movements must be time-resolved, i.e. that the distance a globule moves from one time step to the next cannot be larger than the inter-globule spacing. In our case, an average inter-globule spacing is $2r_{\text{globule}}$ where r is the average radius of the globules in the specimen. In all tracking cases, this is approximately 400 µm. The displacement rate of 5 $\mu m.s^{-1}$ coupled with the 12 s tomograms yield a global movement δ of 60 µm. While an optimal cut-off parameter value of L should be chosen such that $\delta < L < r_{\text{globule}}$ [8], a larger value of L was chosen to deal with inhomogeneities in the deformation and to give the largest chance for all centroids to be tracked. Tracked and labelled globules are shown in Supplementary Figure 12(5), where globules that couldn't be tracked were removed.

Internal porosity. Internal pores were labelled and tracked in the exact manner of the globules. The maximum distance the pore centroids were allowed to move was 30 pixels during each timestep, which is equivalent to 367 µm. While this may seem excessive in comparison to a ram movement of 60 µm, it was necessary in order to compensate for the ability of the pores to move very quickly within the liquid phase during deformation.

Globule translation. Translation and Euler distance of each globule were calculated using the tracked coordinates of each globule centroid. Translation in any given direction was such that, for a globule *g* at time *t* + 1:

$$\begin{aligned} x(g_{t-t+1}) &= x_{t+1} - x_t \\ y(g_{t-t+1}) &= y_{t+1} - y_t \\ z(g_{t-t+1}) &= z_{t+1} - z_t \end{aligned} \qquad \text{Equation (1)}$$

The Euler distance was used as the distance travelled by each globule between time *t* and *t + 1*:

$$d(g_{t-t+1}) = ((x_{t+1} - x_t)^2 + (y_{t+1} - y_t)^2 + (z_{t+1} - z_t)^2)^{1/2} \qquad \text{Equation (2)}$$

Globule rotation. Each globule was cropped and isolated in a 201×201×201 pixel^3 box, which is equivalent to 2.46×2.46×2.46 mm^3. A grain-based image correlation approach was used to obtain the highest normalised correlation coefficient between each globule and its tracked predecessor. This allows each globule to be rotated and displaced in 3D using affine transforms and resulting in a transformation matrix containing rotation, translation and scale components. Converting the rotation matrix to its axis-angle representation permits the rotation component of the transformation matrix to be described as an arbitrary vector around which a rotation of specific angle x takes place. The angle θ, which represents the absolute rotation value of a specific grain in three-dimensional space, is the value of rotation used throughout this work. The process is illustrated in Supplementary Figure 13.

Globule shape. Globule volume and surface area was calculated using a commercially-implemented modified marching cube algorithm.

Sphericity. All mentions of sphericity *S* in this work refer to:

$$S = ((36 \times \pi \times V^2)^{1/3})/S_A \qquad \text{Equation (3)}$$

where V and S_A are the volume and surface area of the globule respectively.

Shell thickness. To determine the extent to which the globules change shape during deformation, 50 globules were selected at random in the experiments where watershed separation was successful. These globules were first registered to their reference globule.

For each registered globule, its shape at one timestep (Supplementary Figure 14(a)) and at the next timestep (Supplementary Figure 14(b)) was added up, the Euclidian distance map of that region was calculated and that distance map was multiplied with the non-overlapping voxels of these datasets (Supplementary Figure 14(c)). In parallel, the overlapping regions of the datasets where dilated by 1 pixel and subtracted from the initial overlapping voxels to produce a ring. The thickness of the shell was obtained by multiplying this ring, which lies at the edge of the overlapping voxel region, with the distance map produced at the earlier step (Supplementary Figure 14(d)).

True axial stress and true axial strain.

Strain. True axial strain was calculated using the height h of the actual specimen as measured in three-dimensions. This distance h was measured in ten slices in the xz direction and averaged, such that the percentage of true strain was given by:

$$\varepsilon_{true} = \ln(h/h_0) \times 100 \qquad \text{Equation (4)}$$

Stress. Load was measured with a 10 Hz frequency continuously during the experiments. Correspondence was established between the first projection of each tomogram and the equivalent 10 load measurements, which were subsequently averaged. This resulted in one load value F representative of the load at the beginning of the capture of each tomogram.

The true stress values used throughout this work were derived directly from the reconstructed images, with the true stress of each tomogram corresponding to the load divided by the measured area in contact with the moving ram.

True volumetric strain. In this work, true volumetric strain is defined in the granular sense. That is to say, with respect to the solid assembly rather than the whole material, such that a contractive volumetric strain occurs if grains move closer together and liquid/gas is expelled, and a dilatational volumetric strain occurs if grains move apart and liquid/gas is drawn into the expanding interstitial spaces It was calculated using the volume formed occupied by the solid assembly, and is calculated as a percentage:

$$\varepsilon_V = \ln(V/V_0) \times 100 \qquad \text{Equation (5)}$$

where watershed grain separation and tracking was possible, the volume formed by the tracked centroids of the globules was computed for each tomogram. The result is a polyhedron with a specific volume forming a hull around the centroids. For very high solid fractions, grains could not be separated using the watershed technique and the volume of the mask obtained was used instead. This was deemed suitable because there is good correlation between volumetric strain calculated using the convex hull of the centroids and the volumetric strain obtained using the convex hull of the specimen edge. This is illustrated in Supplementary Figure 15, where the averaged volumetric strain is plotted. The error bars represent the actual values of the volumetric strain as obtained by both methods for each average. The maximum deviation is 2%, which legitimises the use of the sample edge convex hull as an alternative to centroid convex hull when the centroids cannot be obtained, i.e in the case of samples at very high solid fractions such as 86% and 93% solid.

Supplementary references

1. Yi, J. Z., Gao, Y. X., Lee, P. D., Flower, H. M. & Lindley, T. C. Scatter in fatigue life due to effects of porosity in cast A356-T6 aluminum-silicon alloys. *Metall Mater Trans A* **34**, 1879-1890 (2003).
2. Puncreobutr, C., Lee, P. D., Hamilton, R. W. & Phillion, A. B. Quantitative 3D characterization of solidification structure and defect evolution in Al alloys. *JOM* **64**, 89-95 (2012).
3. Kareh, K. M., Lee, P. D. & Gourlay, C. M. in *MCWASP XIII: International Conference on Modeling of Casting, Welding and Advanced Solidification Processes* Vol. 33 *IOP Conference Series-Materials Science and Engineering* (ed A. Ludwig) (2012).
4. Ganesan, S. & Poirier, D. Densities of aluminum-rich aluminum-copper alloys during solidification. *Metall Trans A* **18**, 721-723 (1987).
5. Titarenko, S., Titarenko, V., Kyrieleis, A., Withers, P. J. & De Carlo, F. Suppression of ring artefacts when tomographing anisotropically attenuating samples. *J Synchrotron Radiat* **18**, 427-435 (2011).
6. Kyrieleis, A., Titarenko, V., Ibison, M., Connolley, T. & Withers, P. J. Region-of-interest tomography using filtered backprojection: assessing the practical limits. *J Microsc* **241**, 69-82 (2011).
7. Studholme, C., Hill, D. L. G. & Hawkes, D. J. An overlap invariant entropy measure of 3D medical image alignment. *Pattern recognition* **32**, 71-86 (1999).
8. Lewis, J. Fast template matching. *Vision Interface* **95**, 120-123 (1995).
